# Ethnomedicinal Plants Used in the Health Care System: Survey of the Mid Hills of Solan District, Himachal Pradesh, India

**DOI:** 10.3390/plants10091842

**Published:** 2021-09-05

**Authors:** Manoj Kumar, Radha, Himani Devi, Suraj Prakash, Sonia Rathore, Mamta Thakur, Sunil Puri, Ashok Pundir, Sneh Punia Bangar, Sushil Changan, Tamilselvan Ilakiya, Mahesh Kumar Samota, Rahul D. Damale, Surinder Singh, Mukesh K. Berwal, Sangram Dhumal, Anilkumar G. Bhoite, Anshu Sharma, Marisennayya Senapathy, Bharat Bhushan, Vineet Kumar Maurya, Asha, Suman Natta, Ryszard Amarowicz, Mohamed Mekhemar

**Affiliations:** 1 Chemical and Biochemical Processing Division, ICAR-Central Institute for Research on Cotton Technology, Mumbai 400019, India; 2 School of Biological and Environmental Sciences, Shoolini University of Biotechnology and Management Sciences, Solan 173229, India; thakurhimani544@gmail.com (H.D.); surajpandiar75@gmail.com (S.P.); sonia369@gmail.com (S.R.); mamtaparmar369@gmail.com (M.T.); sunilpuri@shooliniuniversity.com (S.P.); 3 School of Mechanical and Civil Engineering, Shoolini University of Biotechnology and Management Sciences, Solan 173229, India; ashok.pundir78791@gmail.com; 4 Department of Food, Nutrition and Packaging Sciences, Clemson University, Clemson, SC 29634, USA; snehpunia69@gmail.com; 5 Division of Crop Physiology, Biochemistry and Post-Harvest Technology, ICAR-Central Potato Research Institute, Shimla 171001, India; sushil.changan@icar.gov.in; 6 Department of Vegetable Science, Tamil Nadu Agricultural University, Coimbatore 641003, India; ilakiyatamil@gmail.com; 7 HCP Division, ICAR-Central Institute of Post-Harvest Engineering and Technology, Abohar 152116, India; mahesh.samota@icar.gov.in; 8 ICAR—National Research Centre on Pomegranate, Solapur 413255, India; rahul.damale@icar.gov.in; 9 Dr. S.S. Bhatnagar University Institute of Chemical Engineering and Technology, Panjab University, Chandigarh 160014, India; ssbhinder@pu.ac.in; 10 Division of Crop Improvement, ICAR—Central Institute for Arid Horticulture, Bikaner 334006, India; mukesh.kumar4@icar.gov.in; 11 Division of Horticulture, RCSM College of Agriculture, Kolhapur 416004, India; sangram1326@hotmail.com; 12 Department of Agricultural Botany, RCSM College of Agriculture, Kolhapur 416004, India; anilbhoite5001@gmail.com; 13 Department of Food Science and Technology, Dr. Y.S. Parmar University of Horticulture and Forestry, Nauni 173230, India; anshufst1989@gmail.com; 14 Department of Rural Development and Agricultural Extension, College of Agriculture, Wolaita Sodo University, Wolaita Sodo P.O. Box 138, Ethiopia; drsenapathy@wsu.edu.et; 15 ICAR—Indian Institute of Maize Research, Ludhiana 141004, India; bharat.bhushan@icar.gov.in; 16 Department of Botany and Microbiology, H.N.B. Garhwal University, Srinagar 246174, India; vkmaurya.hnbgu@gmail.com (V.K.M.); ashalko2009@yahoo.com (A.); 17 ICAR—National Research Centre for Orchids, Pakyong 737106, India; nattabiochem@gmail.com; 18 Institute of Animal Reproduction and Food Research, Polish Academy of Sciences, 10-748 Olsztyn, Poland; amaro@pan.olsztyn.pl; 19 Clinic for Conservative Dentistry and Periodontology, School of Dental Medicine, Christian Albrecht’s University, 24105 Kiel, Germany

**Keywords:** ethnobotany, traditional medicine, use value, rural inhabitant

## Abstract

The study was performed in the mid hills of the Dharampur region in Solan district of Himachal Pradesh, India. At the study site, a total of 115 medicinal plants were documented (38 trees, 37 herbs, 34 shrubs, 5 climbers, 1 fern, and 1 grass). In the study region, extensive field surveys were performed between March 2020 and August 2021. Indigenous knowledge of wild medicinal plants was collected through questionnaires, discussions, and personal interviews during field trips. Plants with their correct nomenclature were arranged by botanical name, family, common name, habitat, parts used, routes used, and diseases treated. In the present study, the predominant family was Rosaceae, which represented the maximum number of plant species, 10, followed by Asteraceae and Lamiaceae, which represented 8 plant species. The rural inhabitants of the Dharampur region in the Solan district have been using local plants for primary health care and the treatment of various diseases for a longer time. However, information related to the traditional knowledge of medicinal plants was not documented. The rural inhabitants of the Dharampur region reported that the new generation is not so interested in traditional knowledge of medicinal plants due to modernization in society, so there is an urgent need to document ethnomedicinal plants before such knowledge becomes inaccessible and extinct.

## 1. Introduction

Across the world, rural people rely on traditional local knowledge of medicinal plants for primary health care [1,2]. Ethnobotany is the study of the connection that exists among precolonial cultures of individuals and plant ecosystems. Rural people have inherited traditional medicinal plant knowledge from generation to generation [3,4]. Ethnobotanical studies are essential in the quest for modern drugs derived from natural medicinal plant resources [5,6]. For rural communities in developing countries, the use of plant species as traditional medicines provides a good alternative to health care systems [7,8]. It has been reported that 80% of the population in developing countries relies on traditional medicines for primary health care. These medicinal plants are frequently reported as safe, cheap and easily available from the surroundings [9,10]. In India, approximately 7500 plant species have been reported for medicinal use in indigenous health practices and modern systems of medicines [11,12]. Since Vedic times, plants have been used for medicinal purposes and human sustenance in India. Rig Veda and Atharveda were the first to discuss the medicinal use of plants [13]. In India, approximately 75% of the population lives in rural areas. In periods of food scarcity, most rural communities depend on natural resources such as wild edible plants to meet their food requirements [14].

Approximately 800 species of food plants are eaten by rural communities [15]. Wild plants have had significant importance in human life since ancient times; they have been used for food, medicine, fiber, and other purposes, as well as fodder for livestock. Wild edible food plants are valuable to humans and have been identified as a means of maintaining a balance between population expansion and agricultural productivity, particularly in the developing world [16]. It has been reported that approximately 54 million tribal people live in different parts of India. To continue daily life routines, local inhabitants of rural regions depend on forests and forest products. Most tribal communities still rely on local traditional medicines for their survival [17,18,19].

The biodiversity of the Indian Himalayan region millions of years ago has long been considered an important foundation for traditional medicine [20]. In the northwestern Himalayas, the state of Himachal Pradesh is divided into four zones: dry temperate-alpine, subtropical, subtemperate and moist temperate. The state of Himachal Pradesh has high plant diversity, including endemic and endangered species, due to its varied altitudinal gradients and climatic conditions [21,22]. Ninety-one commercially exploited nontimber forest product species and 57 endangered wild medicinal plant species have been identified by the state forest department of Himachal Pradesh [23]. In some parts of India, there is no proper documentation of ethnomedicinal plants used by rural communities. Therefore, systematic documentation is needed for the conservation of medicinal plant prosperity from rural areas of Himachal Pradesh in India [24,25]. Himachal Pradesh is considered one of the richest areas of traditional and potential medicinal wealth. However, limited studies have been carried out in some regions of the state to document traditional knowledge of ethnomedicinal plants [26,27]

Some researchers have attempted to document useful indigenous information on the medicinal uses of plants from the mid hills of the Dharampur region in Solan district, Himachal Pradesh, India. The survey of this study region can be a good preliminary point for new phytopharmacological research in the medicinal domain. There is no proper record available for traditional medicinal knowledge of plants used by rural people of the mid hills in the Dharampur region of Solan district in Himachal Pradesh, India. With these factors in mind, the present study was carried out with the objective of determining the various uses of medicinal plants. Further studies are required to determine the chemical compounds found in medicinal plants responsible for various biological activities.

## 2. Results

### 2.1. Demography of Informants

Ethnomedicinal data were gathered through open conversations with local informants between the ages of 25 and 75 years. A total of 114 informants, including 76 males (67%) and 38 females (33%), in the study area were interviewed to document their traditional knowledge of ethnomedicinal plants. Based on interviews, it was observed that local males, compared to local females, have better knowledge about ethnomedicines; the reason behind this might be that men are usually favored in the shift of knowledge. However, it is also observed that elderly traditional medicinal practitioners, including both men and women, have equal knowledge about ethnomedicines. In this survey, informants were categorized into five groups based on age. A total of 14 informants were between the ages of 25 and 35, 25 were between the ages of 36 and 50, 30 were between the ages of 51 and 60, 35 were between the ages of 61 and 70, and 10 were between the ages of 70 and 75 years (Table 1).

### 2.2. Ethnomedicinal Plants

A total of 115 ethnomedicinal plants were collected from study site during survey. Table 2 demonstrate botanical name, family, common name (Hindi), habitat, voucher no., part used, administration route, use value and usage.

**Figure 1:**
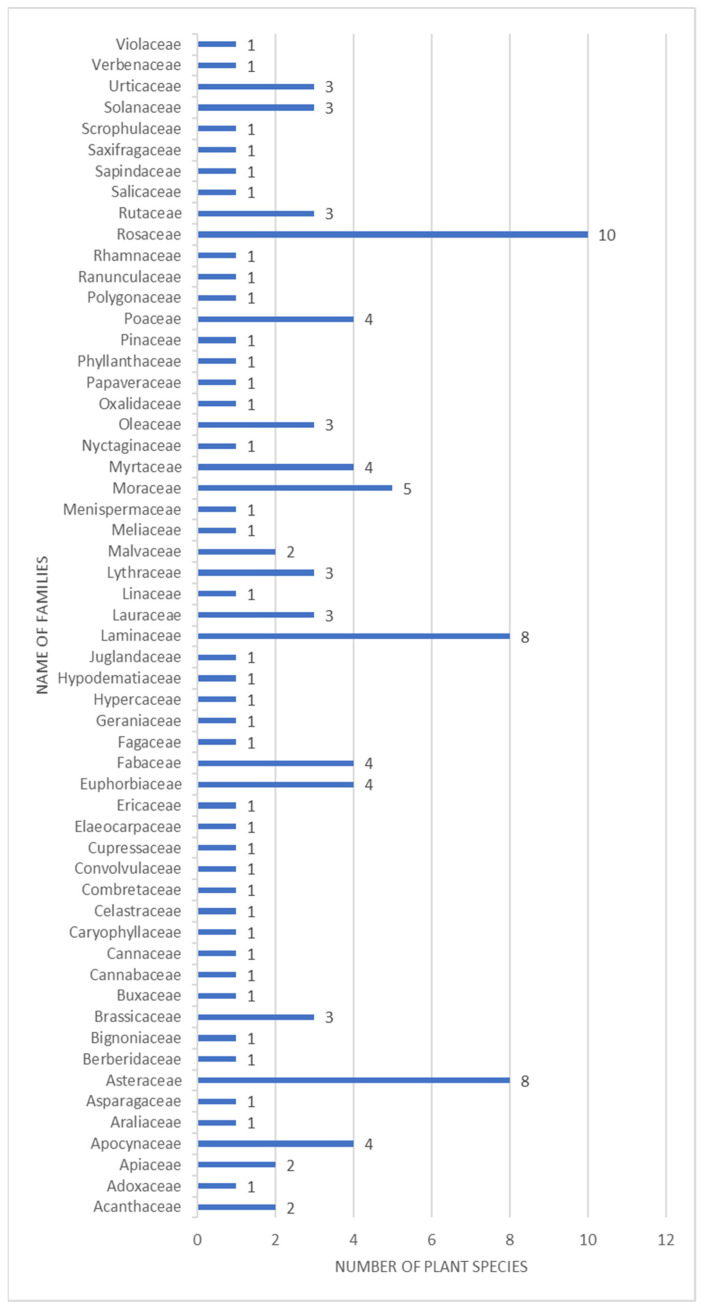
Bar graph showing families and exact number of plants studied during the survey.

The ethnomedicinal plants collected from the study site belongs to families including Apocynaceae, Caryophyllaceae, Asteraceae, Berberidaceae, Brassicaceae, Urticaceae, Elaeocarpaceae, Salicaceae, Polygonaceae, Rhamnaceae, Rosaceae, Sapindaceae and Violaceae, etc. The highest number of ethnomedicinal plants was recorded from the family Rosaceae having 10 plant species followed by Lamiaceae and Asteraceae having 8 plant species (Figure 1).

It was found that all the plants belonging to the Rosaceae are used to cure dysentery, fever, cough, cold and skin diseases, etc. Based on interview data it was observed that skin infection, fever cough and cold occurs more frequently as compared to other diseases. The medicinal plants reported by informers for the remedy of skin infections are *Cryptolepis buchananii*, *Eucalyptus citriodora*, *Ligustrum japonicum*, *Pinus roxburghii*, *Rosa alba*, *Ziziphus nummularia* and *Sonchus oleraceus*.

It was observed that some plants such as *Rhododendron arboreum*, *Zanthoxylum armatum*, *Viola canescens*, *Quercus leucotrichophora*, *Rubus ellipticus*, *Punica granatum*, *Ocimum sanctum*, *Morus nigra*, *Mentha arvensis*, *Justicia adhatoda*, *Ficus benghalensis*, *Eriobotrya japonica*, *Debregeasia longifolia*, *Cissampelos pareira*, *Datura innoxia*, *Eucalyptus citriodora*, *Cynodon dactylon*, *Colebrookea oppositifolia*, and *Cannabis sativa* were suggested by local informants to cure diarrhea, diabetes, dysentery, cough, cold and fever.

**Figure 2:**
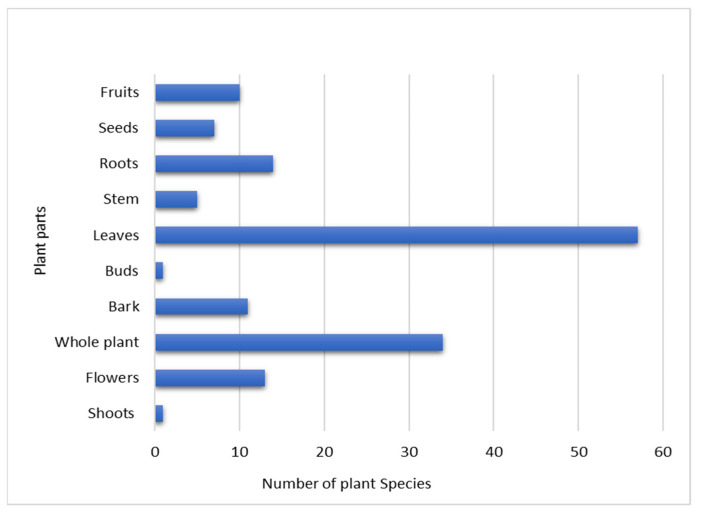
Graph showing the plant parts used for medicinal purposes and the number of plant species studied in the current work.

Based on the informants’ data, leaves were the most commonly used plant part, followed by whole plants, roots and flowers (Figure 2). It was also reported that in the following plant species were used: *Foeniculum vulgare*, *Berberis asiatica*, *Centella asiatica*, *Datura innoxia*, *Elaeocarpus ganitrus*, *Euphorbia heliscopia*, *Euphorbia milii*, *Ipomoea cairica*, *Justicia adhatoda*, *Ligustrum japonicum*, *Nasturtium officinale*, *Mentha arvensis*, *Ocimum sanctum*, *Oxalis corniculata*, *Papaver somniferum*, *Prunus cerasoides*, *Pseudognaphalium hypoleucum*, *Rumex hastatus*, *Punica granatum*, *Ranunculus laetus*, *Salvia officinalis*, *Solanum virum*, *Spiraea cantoniensis* and *Stellaria media*; all plant parts were utilized to cure different diseases. A few medicinal plant species reported from the study site were used in different ritual ceremonies. For example, flowers of *Datura innoxia* and leaves of *Cannabis sativa* are offered to lord Shiva in festivals such as Shivrati, and leaves of *Cynodon dactylon* are offered to lord Ganesh or different deities in Pooja.

### 2.3. Use Value

The results of the ethnobotanical study revealed a wealth of indigenous knowledge and the usage of traditional plants in rural people’s health care systems. The high use value of medicinal plants indicates how important they are to indigenous society in treating specific human ailments. Based on use value data, the most commonly used medicinal plant species is *Catharanthus roseus* (0.90), and the least commonly used medicinal plant species is *Prunus persica* (0.63). *Catharanthus roseus* is used to treat hypertension and diabetes, and *Prunus persica* is used to treat sores and wounds.

**Figure 3:**
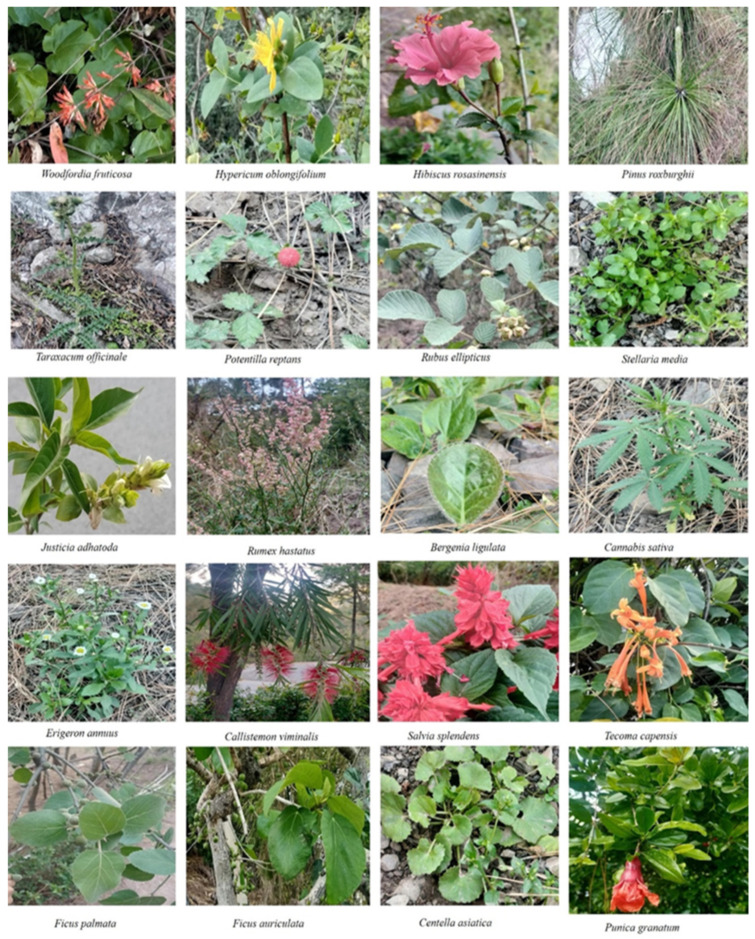

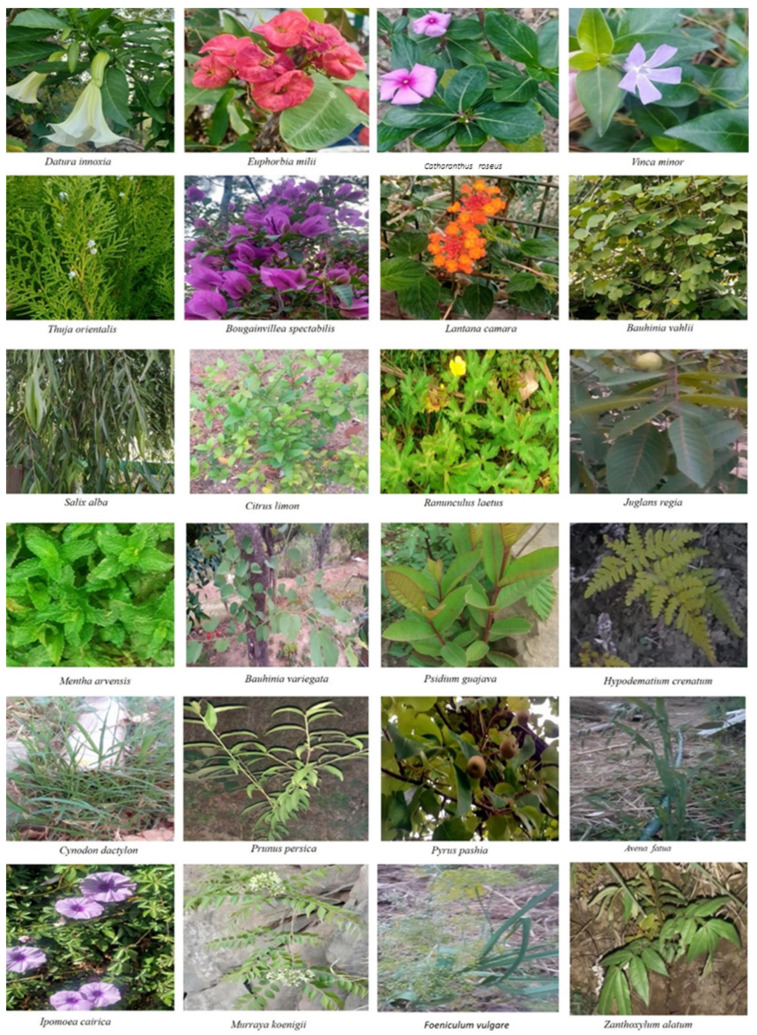

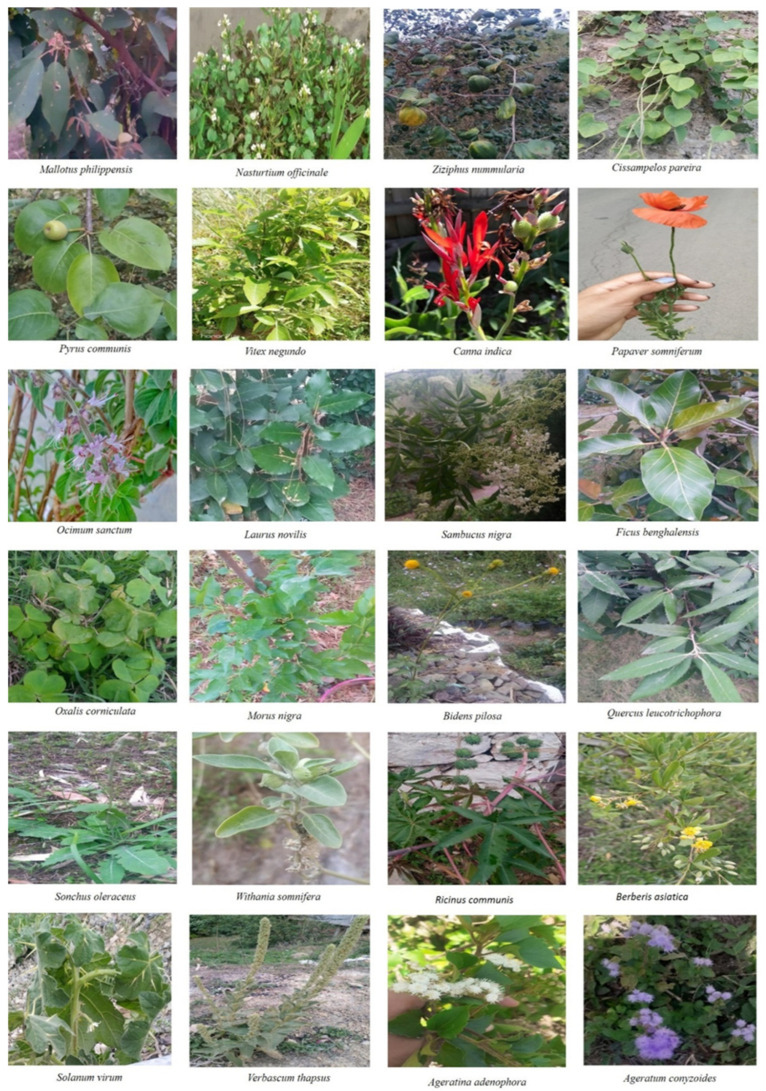
Pictures of medicinal plants surveyed in the current study.

Pictures of some plants reported from the study site are shown in Figure 3.

The ongoing decline of indigenous medicinal plant knowledge requires an assessment of traditional knowledge with the goal of developing the medicinal plant sector. Knowledge on indigenous uses of native plants must be studied before it becomes extinct. The findings of the current study could lead to the development of a new herbal drug for the treatment of ailments. Furthermore, ethnobotanical studies that document indigenous knowledge are important for the conservation and sustainable use of natural resources. It is essential to encourage indigenous groups and enable their participation in sustainable harvesting and conservation of natural resources to implement in situ preservation for traditional knowledge in rural areas. To enhance their position and preserve their knowledge, colleges should engage with indigenous tribes and designate them as “knowledge sites” on technical topics.

Traditional knowledge, biodiversity and cultural values are all interconnected and interdependent. These are, without a doubt, the essential factors that keep traditional knowledge intact in practice. Because of the increasing economic value of biocultural resources and threats to their existence, the government and private entities must recognize these natural resources as national wealth. Governments must establish national policy and legal frameworks to ensure that biocultural resources are effectively protected. The primary goal of this research was to document the ethnomedicinal plants utilized by rural people in the Solan district and to document indigenous knowledge about traditional plant uses through ethnobotanical research. More research into the preparation of medicinal formulations, phytochemicals, and pharmacological significance, followed by clinical trials, will add to the traditional medical and cultural systems’ knowledge base.

## 3. Discussion

In the present study, we documented the uses of commonly used wild medicinal plants in the mid hills of Solan district in Himachal Pradesh, India. A total of 115 plant species belonging to the same or different families were reported from the study region. Due to strong belief in the traditional system of medicine, rural people of the study region frequently prefer to use wild plants. The rural inhabitants of the study site reported that plant species *Berberis aristata*, *Zanthoxylum armatum*, *Viola canescens*, *Rhododendron arboreum*, *Datura innoxia*, *Ocimum sanctum*, *Colebrookea oppositifolia*, *Mentha arvensis*, *Justicia adhatoda*, *Cynodon dactylon*, *Ficus auriculata*, *Cannabis sativa*, *Oxalis corniculata* and *Verbascum thapsus* are highly effective in treating different types of human diseases. A few ethnomedicinal plants found in the current study have also been reported from different regions of India, such as *Verbascum thapsus*, *Cannabis sativa*, *Cynodon dactylon*, *Ficus palmata*, *Urtica dioica* and *Juglans regia*. Most of these formulations were prescribed for oral use. In recent years, it has been reported that traditional ethnobotanical knowledge of medicinal plants is gradually decreasing from society, mainly due to modernization, and some medicinal plants with ethnobotanical importance are threatened with extinction worldwide due to habitat destruction, climate change and overexploitation. Studies have reported that the documentation of ethnobotanical knowledge and ethnomedicinal plants can play a significant role in the conservation of traditional ethnobotanical knowledge and the protection of threatened ethnomedicinal plants [28,29,30,31]. The Himalayan forests provided a rich reservoir of medicinal plants that are essential to the native community [32,33]. Various studies on medicinal plants used by tribal groups in India have found that they prefer traditional medicine since it is less expensive, has fewer side effects, and is a part of their lives and culture on which our findings are set up. In India, some medicinal plants are the only source of health care in remote areas due to a lack of medical facilities [34,35,36,37]. Worldwide, traditional uses of ethnomedicinal plants vary from person to person and region to region [38,39,40,41]. Traditional knowledge of medicinal plants has deteriorated among indigenous groups in recent decades, indicating a risk of extinction. In India, recent economic advancements, exposure to the market economy, and infrastructure modernization have caused a shift in indigenous groups’ traditional lifestyles, resulting in the erosion of traditional knowledge [42]. Using indigenous knowledge of traditional medicine is an efficient method of finding novel medicines through ethnobotanical research. Some of the medicinal plants mentioned in the current study site are also documented in other studies conducted in adjoining regions of Himachal Pradesh. Freshly harvested plants or plant parts are widely utilized in human treatments. Leaves, whole plants, stems, fruits, flowers, seeds, roots, and bark are the most regularly used parts to combat human diseases. Interviewed people were enriched with traditional ethnobotanical knowledge from their parents and grandfathers. It has been found that older people have better traditional knowledge of medicinal plants than younger generations. Ethnobotanical findings could help with the development of indigenous knowledge and its application in domains including pharmacology, pharmacognosy, pharmaceuticals, toxicology, phytochemistry, ethnobotany, taxonomy, anthropology, and medicinal science. This type of alternative medical approach is now recognized as critical for community development [43,44,45]. Recent studies have reported the bioactivities of medicinal plants and phytoextracts, showing potential therapeutic use in the treatment of various ailments. The findings given in this research are preliminary and should be verified further. The link between ethnomedicinal knowledge and modern mainstream pharmacology will be highlighted by pharmacological research on ethnomedicines [46,47,48,49,50]. It has been reported that indigenous knowledge of less-known plants is gradually disappearing [51]. A recent study reported that plant-based treatments may become more popular because of the many negative effects of modern allopathic drugs [52].

Local communities lack proper knowledge of wild plant populations, marketing and selling, inadequate regulation and legal protection and have limited access to appropriate technologies for crop plantation and harvesting. Local communities also require assistance and encouragement to safeguard their knowledge and resources. The rural inhabitants of the study site reported that the new generation is not so interested in traditional knowledge of medicinal plants due to modernization, so there is an urgent need to document traditional knowledge of medicinal plants in the study site before its elimination from society. The current study may be helpful for researchers, teachers, scientists, future generations and different pharmaceutical companies to develop new drugs. A few species of wild medicinal plants (*Berberis aristata*, *Zanthoxylum armatum* and *Viola canescens*) were found to be overexploited by rural people and were illegally collected and sold in markets at high cost. Due to unscientific overexploitation, these plants are found in fewer numbers and need proper maintenance and conservation. Because of the current rapid shift in communities worldwide, ethnobotanical knowledge is at risk. Excessive usage of several wild plants results in destructive harvesting and a loss of plant diversity in the area. Thus, there is a need to raise awareness among the native community about the long-term use and conservation of therapeutic medicines.

## 4. Materials and Methods

### 4.1. Description of the Study Area

**Figure 4:**
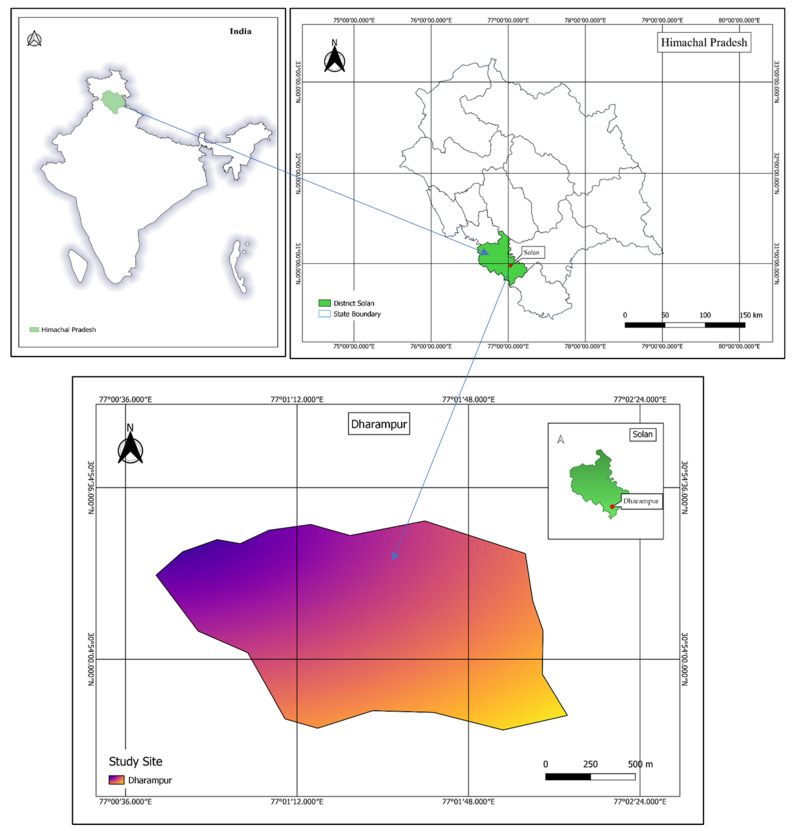
Scale map showing study site Dharampur in the Solan district, Himachal Pradesh, India.

The state of Himachal Pradesh (30°22′40″–33°12′40″ N to 75°45′55″–79°04′20″ E) possesses different types of biodiversity, and it has a pleasant climate throughout the year [53]. The study was performed in the mid hills of Dharampur in Solan district of Himachal Pradesh, India. Solan district is 1350 m above sea level, with a total area of 1936 km^2^. The average annual rainfall is 1413 mm [25]. It snows during the winter season from Jan to Feb. Because of various climatic alterations and altitudinal gradients, Himachal Pradesh is rich in plant biodiversity, including rare and endemic plants [54]. This study documented ethnobotanical knowledge and highlighted medicinal plants that are important in the lives of rural people belonging to the Dharampur region of Solan district. A scale map of the study site is shown in Figure 4.

### 4.2. Data Collection

Extensive field surveys were carried out in the mid hills of the Dharampur region, Solan district, Himachal Pradesh from March 2020 to June 2021, as most of the plants were in the flowering stage and were easy to identify. Ethnobotanical information was gathered through a pretested questionnaire in the format given in the Supplementary information 1 (ethnobotanical survey proforma designed and pretested with local informants, later modified according to the response of informants), direct observation, discussion, and interview methods. It was found that the majority of respondents were between 61 and 75 years old. Informants with better traditional knowledge were selected by the snowball method, and the purpose of the study was explained to informants before they gave oral informed consent. Each informant agreed to participate voluntarily and was allowed to discontinue the interviews any time [55]. Local people served as guides for the field study, and samples of medicinally important plants were collected, with their local identity confirmed by informants. The collected plant specimens were dried and mounted on herbarium sheets with labelled information describing when and how plant samples were collected. All collected plant specimens were identified from the taxonomist of the Botanical Survey of India. Dehradun and voucher specimens were submitted to herbarium of Shoolini University in the Solan district, Himachal Pradesh, India.

### 4.3. Use Value

The importance of plant species was calculated by the use value, and a formula was used for calculation:

*UV* = Σ*U_i_*/*n*

where *U_i_* represents the number of usage reports mentioned by each informant for a particular plant species, and *n* is the total number of informants. If there are many use reports for a plant, the use values are high, which means that the plant is important, and if there are few reports, the use values are low [56,57].

## 5. Conclusions

The current study identified 115 plant species that are utilized to treat a variety of human diseases. The findings of this study show that indigenous people living in remote tribal areas are custodians of knowledge about a wide variety of plant resource uses in the study region. The current study suggests implementing various management strategies with the involvement of indigenous communities through village administrative councils to protect medicinal plants that are threatened by extinction. Ecology is shaped by the dialectical relationship between indigenous knowledge and practice, which has an impact on the plant population. New hypotheses for sustainable resource conservation can be developed by combining indigenous knowledge and use in scientific study. Indigenous knowledge of plant resource utilization is constantly decreasing due to changing perceptions of local people and the ever-increasing influence of globalization and socioeconomic transformation. The amount of valuable plant resources is diminishing at an alarming rate due to a lack of controlled scientific and sustainable monitoring cultivation and harvesting, lack of proper management techniques, and lack of knowledge of social concerns. Additionally, indigenous knowledge of lesser-known plant uses is rapidly disappearing. Plant-based treatments may become more popular as a result of the many negative effects of modern allopathic drugs, and traditional knowledge of plants and folk remedies may be preserved. Rural inhabitants of the study area reported that the new generation is not so interested in traditional knowledge of medicinal plants due to Western influence in society, so there is an urgent need to document traditional knowledge of medicinal plants from the study region of Solan district in Himachal Pradesh. A new generation may become more aware of natural products and motivated to utilize them. However, there is less information on the active phytochemicals in these plant species; therefore, the active principles responsible for pharmacological action must be investigated further at a scientific level to validate the claim.

## Figures and Tables

**Table 1:** People of different age groups were randomly selected for interviews.

Sr. No.	Age Group	Male	Female
1	25–35	10 (13.1%)	4 (10.52%)
2	36–50	19 (25%)	6 (15.78%)
3	51–60	20 (26.31%)	10 (26.31%)
4	61–70	21 (27.63%)	14 (36.84%)
5	70–75	6 (7.89%)	4 (10.52%)
Education level	Male	Female	
Illiterate	19 (25%)	8 (21.05%)	
Primary school level	14 (18.42%)	14 (36.84%)	
Secondary school level	21 (27.63%)	11 (28.94%)	
High school level	13 (17.10%)	3 (7.89%)	
Graduate	9 (11.84%)	2 (5.26%)	

Sr. No.—serial number.

**Table 2:** Ethnobotanical data documented from the study site.

Sr. No.	Botanical Name	Family	Common Name (Hindi)	Habit	Voucher No.	Part Used	Administration Route	Use Value (UV)	Usage
1	*Ageratina adenophora* (Spreng.) R.M. King and H. Rob	Asteraceae	Banmara	Shrub	SUBMS/BOT-3901	Leaves	Topical	0.13	Leaf extract is used to treat cuts and wounds.
2	*Ageratum conyzoides* L.	Asteraceae	Ghabuti	Herb	SUBMS/BOT-3902	Leaves, Roots	Topical	0.59	A paste made from the leaves is used as a wrapping to remove spines from the skin. Juice of leaves and roots is used to treat cuts and wounds.
3	*Ajuga integrifolia* Buch.—Ham.	Lamiaceae	Neelkanthi	Herb	SUBMS/BOT-3903	Whole plant	Oral	0.25	Whole plant is used to treat diarrhea.
4	*Asparagus racemosus* Willd.	Asparagaceae	Shatavari	Herb	SUBMS/BOT-3904	Roots, Leaves	Oral	0.30	The leaves and roots extract are used to treat kidney disorders, fevers, stomach ulcer and diarrhea.
5	*Avena fatua* L.	Poaceae	Joa	Herb	SUBMS/BOT-3905	Seeds	Oral	0.11	Seeds are used to treat itchy skin and ulcers.
6	*Bambusa vulgaris* Schrad.	Poaceae	Bans	Tree	SUBMS/BOT-3906	Shoots, Bark	Oral	0.27	Shoot powder is boiled with hot water and used against malaria. The bark powder is boiled with hot water to stimulate blood flow in pelvic area especially during menstruation.
7	*Bauhinia vahlii* Wight and Arn.	Fabaceae	Torre	Climber	SUBMS/BOT-3907	Leaves	Oral	0.31	Juice extracted from the leaves is used to kill stomach worms.
8	*Bauhinia variegata* L.	Fabaceae	Kachnar	Tree	SUBMS/BOT-3908	Roots, Buds	Oral	0.37	Roots are used to prepare antidote to snake poisoning. Dried buds are used during dysentery.
9	*Berberis asiatica* Roxb. Ex DC.	Berberidaceae	Kashmal	Shrub	SUBMS/BOT-3909	Whole plant	Topical	0.85	Whole plant paste is used to apply on wounds and cuts.
10	*Bergenia ligulata* Engl.	Saxifragaceae	Dakachru	Herb	SUBMS/BOT-3910	Whole plant	Oral	0.43	Decoction of whole plant is used for urinary infection or kidney stones.
11	*Bidens pilosa* L.	Asteraceae	Gumber	Herb	SUBMS/BOT-3911	Leaves	Oral	0.33	Decoction of leaves is used to treat constipation, fever and diabetes.
12	*Boehmeria platyphylla* D. Don	Urticaceae	Handa	Shrub	SUBMS/BOT-3912	Leaves	Oral	0.10	The leaves are used to treat bleeding gums.
13	*Bougainvillea spectabilis* Willd.	Nyctaginaceae	Booganbel	Shrub	SUBMS/BOT-3913	Whole plant	Oral	0.18	Whole plant is helpful in the treatment of diabetes.
14	*Buxus sempervirens* L.	Buxaceae	-	Shrub	SUBMS/BOT-3914	Leaves	Oral	0.05	The leaves are used in the treatment of malaria.
15	*Callistemon viminalis* (Sol. Ex Gaertn.) G.Don	Myrtaceae	Cheel	Tree	SUBMS/BOT-3915	Whole plant	Oral	0.06	Whole plant is used for the treatment of diarrhea, skin infection and urinary infections.
16	*Canna indica* L.	Cannaceae	Sarvajjaya	Herb	SUBMS/BOT-3916	Seeds	Topical	0.26	The seed paste is used to treat fever.
17	*Cannabis sativa* L.	Cannabaceae	Bhang	Herb	SUBMS/BOT-3917	Seeds, Leaves	Oral, Topical	0.69	Seeds are used to treat asthma and relief from body pain. The leaves extract is used to treat cuts, burn, diabetes and dysentery.
18	*Carissa spinarum* L.	Apocynaceae	Garna	Shrub	SUBMS/BOT-3918	Fruits	Oral	0.44	Fruit extract is used to treat fever, diarrhea and toothache.
19	*Catharanthus roseus* (L.) G. Don	Apocynaceae	Sadabahar	Shrub	SUBMS/BOT-3919	Roots, Leaves	Oral	0.90	Decoction of roots and leaves are used to treat hypertension and diabetes.
20	*Centella asiatica* (L.) Urb.	Apiaceae	Brahmi	Herb	SUBMS/BOT-3920	Leaves	Oral	0.52	The leaves are taken with sugar as memory enhancer.
21	*Cinnamomum camphora* (L.) J. Presl	Lauraceae	Kapur	Tree	SUBMS/BOT-3921	Whole plant	Oral	0.39	Whole plant is used to treat cough, cold, skin irritation and low blood pressure.
22	*Cissampelos pareira* L.	Menispermaceae	Batindu	Climber	SUBMS/BOT-3922	Leaves, Stem	Oral	0.11	Infusion of leaves and stem is used to treat diarrhea, dysentery and digestive complaints.
23	*Citrus limon* (L.) Osbeck	Rutaceae	Nimbu	Tree	SUBMS/BOT-3923	Fruits	Oral	0.34	Fruit juice is taken orally for indigestion.
24	*Clinopodium vulgare* L.	Lamiaceae	Jangalee tulsi	Herb	SUBMS/BOT-3924	Leaves	Topical	0.22	The leaves are used to treat wounds and cuts.
25	*Colebrookea oppositifolia* (Smith.)	Lamiaceae	Gaddoos	Shrub	SUBMS/BOT-3925	Leaves, Stem	Oral, Topical	0.27	Stem is used for cough. Leaf paste is used to treat wounds and eye infection.
26	*Coronopus didymus* (L.) Smith	Brassicaceae	Garhbini	Herb	SUBMS/BOT-3926	Leaves	Oral	0.19	The leaves are used to treat asthma.
27	*Cryptolepis buchananii* Roem. and Schult.	Apocynaceae	Kala bel	Climber	SUBMS/BOT-3927	Roots, Stem	Oral	0.23	Roots are used to treat loss of appetite, fever, skin infections and considered as blood purifier. Stem is used for the treatment of inflammation, muscle and joint pain.
28	*Cynodon dactylon* (L.) Pers.	Poaceae	Drub	Grass	SUBMS/BOT-3928	Leaves	Oral	0.50	The leaves are used to treat cough, cancer, diarrhea, dysentery and hypertension.
29	*Datura innoxia* Mill.	Solanaceae	Datura	Shrub	SUBMS/BOT-3929	Whole plant	Oral	0.44	Whole plant is used to treat fever, diarrhea, cold, asthma and relief body pain.
30	*Debregeasia longifolia* (Burm. f.) Wedd.	Urticaceae	Sansaru	Shrub	SUBMS/BOT-3930	Leaves	Oral	0.04	The leaves are used to treat dysentery and indigestion.
31	*Dicliptera bupleuroides* Nees	Acanthaceae	Kuthi	Herb	SUBMS/BOT-3931	Leaves	Topical	0.07	The leaves are warmed and kept on joints to relieve pains.
32	*Elaeocarpus ganitrus* Roxb.	Elaeocarpaceae	Rudraksha	Tree	SUBMS/BOT-3932	Whole plant	Oral	0.13	Whole plant is used to treat mental illness, cough and hepatic diseases.
33	*Erigeron annuus* (L.) Pers.	Asteraceae	Phuntha	Herb	SUBMS/BOT-3933	Leaves	Oral	0.18	The leaves extract is used to treat diabetes.
34	*Eriobotrya japonica* (Thunb.) Lindl.	Rosaceae	Lokat	Tree	SUBMS/BOT-3934	Leaves, Fruits	Oral	0.37	Decoction of the leaves is used to treat cough and cold. Fruits are used to relieve vomiting and thirst.
35	*Eruca vesicaria* (L.) Cav	Brassicaceae	Tara mira	Herb	SUBMS/BOT-3935	Leaves	Oral	0.18	The leaves are used to treat diarrhea.
36	*Eucalyptus citriodora* Hook.	Myrtaceae	Safeda	Tree	SUBMS/BOT-3936	Leaves	Oral, Topical	0.14	Leaves are used to treatcough, cold, sore throat, cuts and skin infections.
37	*Euonymus tingens* Wall.	Celastraceae	Barmeli	Tree	SUBMS/BOT-3937	Bark	Oral	0.10	The juice of the bark is used in the treatment of eye diseases.
38	*Euphorbia helioscopia* L.	Euphorbiaceae	Dudhi	Herb	SUBMS/BOT-3938	Whole plant	Oral, Topical	0.37	Paste of the plant applied for healing wounds. Milky latex is applied externally on skin to treat fungal infection.
39	*Euphorbia milii* Var- splenden	Euphorbiaceae	-	Shrub	SUBMS/BOT-3939	Whole plant	Topical	0.08	Whole plant is widely used in folk medicine for the treatment of cancer and hepatitis.
40	*Ficus auriculata* Lour.	Moraceae	Tiamble	Tree	SUBMS/BOT-3940	Stem, Fruits	Oral, Topical	0.34	The latex from the stems is applied to cuts and wounds. The roasted fruits are used in the treatment of diarrhea and dysentery.
41	*Ficus benghalensis* L.	Moraceae	Bargad	Tree	SUBMS/BOT-3941	Leaves	Oral	0.30	The leaves are used to treat dysentery and diarrhea.
42	*Ficus palmata* Forssk.	Moraceae	Fagura	Tree	SUBMS/BOT-3942	Fruits	oral	0.37	The fruits are used to treat constipation.
43	*Ficus religiosa* L.	Moraceae	Pipal	Tree	SUBMS/BOT-3943	Bark, Roots	Oral, Topical	0.21	Decoction of the bark is used to control diabetes Roots is used to treat joint swellings.
44	*Foeniculum vulgare* Gaertn.	Apiaceae	Saunf	Herb	SUBMS/BOT-3944	Whole plant	Oral, Topical	0.55	Infusion of whole plant parts is used to treat stomach pain and kidney stones. The leaves paste is applied to healing wounds and skin rashes.
45	*Geranium wallichianum* D. Don ex Sweet	Geraniaceae	Sucha Phulli	Herb	SUBMS/BOT-3945	Roots	Oral, Topical	0.22	Roots are chewed to stop gum bleeding. Decoction of roots is used to treat kidney stones.
46	*Hedera nepalensis* K. Koch.	Araliaceae	Bano	Climber	SUBMS/BOT-3946	Leaves	Oral	0.28	The leaves are used to treat diabetes and skin infections.
47	*Hibiscus rosasinensis* L.	Malvaceae	Gurhal	Tree	SUBMS/BOT-3947	Flowers, Leaves, Roots	Oral, Topical	0.68	Flowers are used in the treatment of excessive and painful menstruation. Decoction of leaves are used as a lotion in the treatment of fever. Decoction of roots is used to treat sore eyes.
48	*Hypericum oblongifolium* choisy	Hypercaceae	Basant	Shrub	SUBMS/BOT-3948	Leaves	Oral, Topical	0.31	Leaves extract is used for wounds and juice as an antidote against snakebite.
49	*Hypodematium crenatum* (Forssk.) Kunh	Hypodematiaceae	Jadi buti	Fern	SUBMS/BOT-3949	Leaves	Topical	0.06	The leaves are used to treat constipation.
50	*Indigofera heterantha* Brandis	Fabaceae	Kali-kathi	Shrub	SUBMS/BOT-3950	Flowers	Oral	0.21	Flowers are used in the treatment of abdominal pain and liver infection.
51	*Ipomoea cairica* (L.) Sweet	Convolvulaceae	Neeli Bel	Climber	SUBMS/BOT-3951	Whole plant	Oral	0.31	Whole plant is used to treat jaundice, fever, and liver infection.
52	*Jasminum sambac* (L.) Aiton	Oleaceae	Mogra	Shrub	SUBMS/BOT-3952	Flowers, Leaves	Oral, Topical	0.11	Flowers are used to treat jaundice, ulcers, boils, and eye infections. Leaves are used to treat wounds.
53	*Juglans regia* L.	Juglandaceae	Akhrot	Tree	SUBMS/BOT-3953	Leaves, Bark	Oral, Topical	0.77	Decoction of the leaves are used to treat skin diseases like scabies and ringworm. Paste of the bark is applied to treat fresh wounds and toothache.
54	*Justicia adhatoda* L.	Acanthaceae	Arusa	Shrub	SUBMS/BOT-3954	Whole plant	Oral	0.23	Whole plant is used to treat cough, cold and asthma.
55	*Koelreuteria paniculate* Laxm.	Sapindaceae	-	Tree	SUBMS/BOT-3955	Flowers	Oral	0.07	Flowers are used in the treatment of conjunctivitis.
56	*Lagerstroemia indica* L.	Lythraceae	Sawani	Tree	SUBMS/BOT-3956	Flowers, Roots	Oral, Topical	0.13	Paste of the flowers is applied to treat cuts and wounds. Decoction of the root is used in the treatment of cold.
57	*Lantana camara* L.	Verbenaceae	Raimuniya	Shrub	SUBMS/BOT-3957	Whole plant	Oral	0.33	Whole plant is used to treat cough, headache, constipation.
58	*Lathyrus aphaca* L.	Fabaceae	Jangli mattar	Herb	SUBMS/BOT-3958	Seeds	Oral	0.32	Seeds are used in the treatment of toothache.
59	*Laurus nobilis* L.	Lauraceae	Tej patta	Tree	SUBMS/BOT-3959	Leaves	Oral	0.62	Decoction of the leaves are used to treat urinary infection.
60	*Ligustrum japonicum* Thunb.	Oleaceae	-	Tree	SUBMS/BOT-3960	Whole plant	Oral	0.08	Extract of whole plant is used to treat ulcer and skin infections.
61	*Machilus duthei* King	Lauraceae	-	Tree	SUBMS/BOT-3961	Leaves	Topical	0.04	The leaves are used to cure pimples.
62	*Malloyus philippensis* (Lam.)	Eurphorbiaceae	Kamala	Tree	SUBMS/BOT-3962	Bark, Leaves	Oral, Topical	0.59	Bark is used to treat stomach ulcers. Decoction of the leaves is used to treat diarrhea.
63	*Malvastrum coromandelianum* (L.) Garcke	Malvaceae	Kharenti	Herb	SUBMS/BOT-3963	Leaves	Oral, Topical	0.08	The leaves paste applied for healing wounds.
64	*Melia azedarach* L.	Meliaceae	Bakain	Tree	SUBMS/BOT-3964	Leaves, Flowers	Oral, Topical	0.14	The flowers and leaves are used to treat headache.
65	*Mentha arvensis* L.	Lamiaceae	Pudina	Herb	SUBMS/BOT-3965	Whole plant	Oral	0.68	Whole plant is used to treat fever, headache and stomach diseases.
66	*Morus nigra* L.	Moraceae	Tut	Tree	SUBMS/BOT-3966	Leaves, Roots	Oral	0.34	The leaves are used to treat cold and eye infections. Roots are used to treat asthma, coughs, hypertension and diabetes.
67	*Murraya koenigii* (L.) Spreng.	Rutaceae	Kari patta	Shrub	SUBMS/BOT-3967	Leaves	Oral, Topical	0.79	The leaves extract is used to treat diabetes and indigestion.
68	*Nasturtium officinale* R. Br.	Brassicaceae	Jal-indushoor	Herb	SUBMS/BOT-3968	Whole plant	Oral, Topical	0.08	The freshly prepared juice of whole plant is used to treat chest infection.
69	*Ocimum sanctum* L.	Lamiaceae	Tulsi	Shrub	SUBMS/BOT-3969	Whole plant	Oral	0.88	Whole plant is used to treat asthma, malaria, diarrhea, dysentery, eye diseases and insect bite.
70	*Olea europaea* L.	Oleaceae	Kahu	Tree	SUBMS/BOT-3970	Leaves, Fruits	Oral	0.33	Decoction of leaves and fruits are used to treat diarrhea, respiratory infections and urinary tract infections.
71	*Oxalis corniculata* L.	Oxalidaceae	Amrul	Herb	SUBMS/BOT-3971	Whole plant	Topical	0.21	The juice of whole plant is used to treat muscular swellings, boils and pimples.
72	*Papaver somniferum* L.	Papaveraceae	Afim	Herb	SUBMS/BOT-3972	Whole plant	Oral	0.61	Infusion of whole plant juice is used to treat fever, cough and headache.
73	*Phyllanthus emblica* L.	Phyllanthaceae	Amla	Tree	SUBMS/BOT-3973	Fruits	Oral	0.76	Fruit juice is used to treat diarrhea, jaundice, diabetes and inflammation.
74	*Pinus roxburghii* Sarg.	Pinaceae	Chir	Tree	SUBMS/BOT-3974	Leaves, Bark, Roots	Oral, Topical	0.66	Bark paste is used in burns, cracks, skin infections and ulcers. Leaves are used to treat fever. Root extract is used to treat eye infections.
75	*Potentilla indica* (Jacks.) Th. Wolf.	Rosaceae	kiphaliya	Herb	SUBMS/BOT-3975	Leaves	Topical	0.21	The leaves are used to treat boils, burns and snake bites.
76	*Potentilla nepalensis* L.	Rosaceae	Ratanjot	Herb	SUBMS/BOT-3976	Leaves, Stem	Oral	0.22	Decoction of leaves and stem are used to treat inflammation of the body and joints.
77	*Prunus cerasoides* Buch.-Ham.	Rosaceae	Padam	Tree	SUBMS/BOT-3977	Whole plant	Oral	0.43	Whole plant is used to treat skin infections and renal stones.
78	*Prunus persica* (L.) Batsch	Rosaceae	Aru	Tree	SUBMS/BOT-3978	Leaves, Flowers	Oral, Topical	0.63	The leaves paste is used to treat sores and wounds. Flowers are used to treat constipation.
79	*Pseudognaphalium hypoleucum* (DC.) Hilliard and B.L. Burtt	Asteraceae	Goiphul	Herb	SUBMS/BOT-3979	Whole plant	Oral	0.11	Whole plant is used for the treatment of cough and body pain.
80	*Psidium guajava* L.	Myrtaceous	Amrood	Tree	SUBMS/BOT-3980	Leaves, Fruits	Oral	0.60	Leaves are used to treat diarrhea. Fruits are used to treat cough, and oral ulcers.
81	*Punica granatum* L.	Lythraceae	Anar	Shrub	SUBMS/BOT-3981	Whole pant	Oral	0.55	Whole plant is used in the treatment of dysentery, stomach-ache, jaundice and diarrhea.
82	*Pyrus communis* L.	Rosaceae	Nashpati	Tree	SUBMS/BOT-3982	Leaves, Bark	Oral, Topical	0.83	The leaves are used to treat inflammation. Decoction of bark is used to treat sprains.
83	*Pyrus pashia* Buch- Hum.	Rosaceae	Shegal	Tree	SUBMS/BOT-3983	Leaves, Flowers	Oral, Topical	0.75	The leaves are used to treat sores and wounds. Flowers are used as internally in the treatment of constipation.
84	*Quercus leucotrichophora* A. Camus	Fagaceae	Ban	Tree	SUBMS/BOT-3984	Seeds	Oral	0.50	Seed decoction is used to treat dysentery and diarrhea.
85	*Ranunculus laetus* Wall. Ex Hook.f. and J.W. Thomson	Ranunculaceae	Jaldhaniya	Herb	SUBMS/BOT-3985	Whole plant	Oral	0.44	Whole plant is used in the treatment of fever and asthma.
86	*Reinwardita indica* (Dumort.)	Linaceae	Basanti	Shrub	SUBMS/BOT-3986	Whole plant	Topical	0.24	Whole plant is used to treat cuts, wounds and boils.
87	*Rhododendron arboreum* Smith.	Ericaceae	Burans	Shrub	SUBMS/BOT-3987	Leaves, Flowers	Oral	0.77	The leaves are used to treat headache, cough, diarrhea and dysentery. Juice of flower is used to treat menstrual disorders.
88	*Ricinus communis* L.	Euphorbiaceae	Arandi	Shrub	SUBMS/BOT-3988	Leaves	Topical	0.23	The leaves are used to treat cuts, swollen joints, inflammation and liver disorders.
89	*Rosa alba* L.	Rosaceae	Gulab	Shrub	SUBMS/BOT-3989	Flowers	Topical	0.37	Flowers are used to treat skin infections.
90	*Rubus ellipticus* Smith	Rosaceae	Aakhae	Shrub	SUBMS/BOT-3990	Roots, Fruit	Oral	0.86	Root extract is used to cure headaches and stomach pain. Fruit juice is used to cure cough, fever and dysentery.
91	*Rumex hastatus* D. Don	Polygonaceae	Khattib-uti	Shrub	SUBMS/BOT-3991	Whole plant	Oral	0.50	Whole plant is used to treat indigestion, skin diseases and constipation.
92	*Salix alba* L.	Salicaceae	Bains	Tree	SUBMS/BOT-3992	Bark	Oral	0.31	Bark is used as a remedy for cold, fevers and joint pain.
93	*Salvia officinalis* L.	Lamiaceae	Sage	Shrub	SUBMS/BOT-3993	Whole plant	Oral, Topical	0.28	Whole plant is used to treat insect bites, gum infections and vaginal discharge.
94	*Salvia splendens* Sellow ex Schult	Lamiaceae	Salbia sefakuss	Herb	SUBMS/BOT-3994	Leaves, Seeds	Oral, Topical	0.08	The leaves are used for dressing wounds, cold, cough and diabetes. Seeds are used to treat dysentery.
95	*Sambucus nigra* L.	Adoxaceae	Berry	Tree	SUBMS/BOT-3995	Flower, Fruits	Oral	0.38	Extracts of the flowers and fruits are used to treat cold. Fruits are used to treat headaches, dental pain, chest pain and nerve pain.
96	*Setaria viridis* (L.) P.Beauv.	Poaceae	Makriya	Herb	SUBMS/BOT-3996	Seeds, Leaves	Oral	0.27	The seed is diuretic and used to treat fever. The leaves are crushed and mixed with water then used to treat wounds and cuts.
97	*Solanum virum* Dunal	Solanaceae	Kandiyari	Shrub	SUBMS/BOT-3997	Whole plant	Oral	0.32	The whole plant is used to treat headaches, indigestion and stomach diseases.
98	*Sonchus oleraceus* L.	Asteraceae	Dudhi	Herb	SUBMS/BOT-3998	Leaves	Oral	0.43	The plant leaves are used to treat inflammatory swellings and skin diseases.
99	*Spiraea cantoniensis* Lour.	Rosaceae	Jhar mairala	Shrub	SUBMS/BOT-3999	Whole plant	Oral	0.37	Decoction of whole plant is used to treat skin infection.
100	*Stellaria media* L. Vill.	Caryophyllaceae	Buch-bucha	Herb	SUBMS/BOT-4000	Whole plant	Oral, Topical	0.55	Whole plant is used to heal skin wound, treat itchiness, indigestion, asthma and respiratory problems.
101	*Syzygium cumini* (L.) Skeels	Myrtaceae	Jamun	Tree	SUBMS/BOT-4001	Bark, Leaves	Oral, Topical	0.44	Juice of bark is used to treat wounds and enlargement of the spleen. Leaves are used to treat diabetes and diarrhea.
102	*Tagetes erecta* L.	Asteraceae	Genda	Herb	SUBMS/BOT-4002	Leaves, Flowers	Oral, Topical	0.25	Decoction of flowers is used to treat cold and mumps. Leaves paste is applied externally to treat skin diseases and conjunctivitis.
103	*Taraxacum officinale* L.	Asteraceae	-	Herb	SUBMS/BOT-4003	Whole plant	Oral	0.09	The whole plant is used for indigestion and jaundice.
104	*Tecoma capensis* (Thunb.) Lindl.	Bignoniaceae	-	Shrub	SUBMS/BOT-4004	Bark, Leaves	Oral	0.07	Bark powder is used to treat fever, pneumonia and stomach troubles. Leaves are used to treat diarrhea and intestinal inflammation.
105	*Terminalia arjuna* (Roxb. Ex DC.) Wight and Arn	Combretaceae	Arjun	Tree	SUBMS/BOT-4005	Bark	Oral	0.37	Bark extract used to treat dysentery, anemia and asthma.
106	*Thuja orientalis* L.	Cupressaceae	Morpankhi	Tree	SUBMS/BOT-4006	Leaves	Oral	0.33	The leaves are used to treat skin infections.
107	*Urtica dioica* L.	Urticaceae	Kuksh	Herb	SUBMS/BOT-4007	Whole plant	Oral	0.55	Whole plant is used to treat kidney stones and skin disorders.
108	*Verbascum thapsus* L.	Scrophulariaceae	Tamakhu	Herb	SUBMS/BOT-4008	Leaves, Flowers	Oral	0.85	Juice of leaves is used to treat fever. Flowers are used to treat cough.
109	*Vinca minor* L.	Apocynaceae	-	Herb	SUBMS/BOT-4009	Leaves, Roots	oral	0.44	The leaves are used to treat internal injury, heavy menstrual bleeding and nose bleeding. Root is used to reduce the blood pressure.
110	*Viola canescens* wall.	Violaceae	Banafsha	Herb	SUBMS/BOT-4010	Whole plant	Oral	0.84	Whole plant is used to treat asthma and cold.
111	*Vitex negundo* L.	Lamiaceae	Bana	Shrub	SUBMS/BOT-4011	Leaves	Topical	0.81	Smoke of leaves is inhaled to get rid of cough.
112	*Withania somnifera* L.	Solanaceae	Ashwagandha	Shrub	SUBMS/BOT-4012	Roots, Leaves	Oral, Topical	0.55	Roots are used to treat inflammation of joints, rheumatic pain, cold, cough and ulcers. Leaves are applied for inflammation and swelling.
113	*Woodfordia fruiticosa* (L.) kurz	Lythraceae	Dhawai	Shrub	SUBMS/BOT-4013	Whole plant	Oral, Topical	0.78	Whole plant is used in the treatment of dysentery and skin diseases.
114	*Zanthoxylum armatum* DC.	Rutaceae	Tirmir	Shrub	SUBMS/BOT-4014	Whole plant	Oral, Topical	0.80	Whole plant is used to treat asthma, diarrhoea, cold, fever, cough, toothache and indigestion.
115	*Ziziphus nummularia* (Burm. f.) Wight and Arn.	Rhamnaceae	Ber	Shrub	SUBMS/BOT-4015	Leaves	Topical	0.78	The leaves are used to treat skin infections.

## Data Availability

Data will be available on request.

## Supplementary Materials

The following are available online at https://www.mdpi.com/article/10.3390/plants10091842/s1, Supplementary information 1: Questionnaire for conducting the ethnomedicinal study.
