# Supplementary material for: Ethnomedicinal Plants Used in the Health Care System: Survey of the Mid Hills of Solan District, Himachal Pradesh, India
Source: Plants (Basel). 2021 Sep 5;10(9):1842. doi: 10.3390/plants10091842 (PMC8467016; doi:10.3390/plants10091842)
Supplement: Supplementary file 1 [file plants-10-01842-s001.zip › plants-1323992-supplementary.pdf]

plants MDPI

Supplementary information 1

Questionnaire for conducting the ethnomedicinal study

(A) **Demographic data**

Name of Tehsil......................
Name of Village....................
Rural ....
Address…………………….
Telephone……………………
Sr. No……………….......Age………………. Education…………………….
1.
2.

(B) **Questions for informants**

1. For how long you have been traditional medicine practitioner?
2. Which plant or plant product have you used for medicinal purposes?
3. Which part of plant do you use?
4. How is it used? (Fresh or Dried)
5. How is the preparation administered?

(C) **Ethnomedicinal plant uses**

1. Plant (Local / Vernacular name)
2. Plant identified as.. (Botanical name)
3. Habit of the plant (Trees/Shrubs/Herbs/Climbers/Grasses/Other)
4. Part(s) of plant used...
5. Nature of ailment treated...
6. Route of administration (a) Oral (b) Topical.
7. Response of the informant(s).
(a) Effective/Good................. (b) Fair................ (c) Poor.............

(D) **Informant's declaration**

We, the above-mentioned, have voluntarily agreed to participate in this study with our full consent, and we declare that the information and knowledge given in the interview and discussion is correct and complete to the best of our knowledge.

Dated: ....
